# On an Improved Test for Detecting Sugar in the Urine

**Published:** 1874-12-01

**Authors:** W. S. Haines


### ON AN IMPROVED TEST FOR DETECTING SUGAR IN THE URINE.

By W. S. Haines, M.D.

WITHOUT doubt, the best method that we possess, at the present time, for detecting the presence of sugar in the urine, for clinical purposes, is by the use of what is known as Fehling's test,—its simplicity, accuracy, and ease of application rendering it by far the most desirable of all the many tests that have been suggested. Unfortunately, however, associated with all these desirable features there are two serious objections, soon to be mentioned, which very materially limit the field of its usefulness and prevent the great majority of the profession enjoying the advantages of its excellence. We believe, therefore, that certain suggestions which we are about to propose, in the way of modifying this test, so that its objectionable features are entirely removed while its many advantages remain wholly unchanged, will be found of value to the profession.

Fehling's test, as now usually employed, consists in making up, in the first place, a test solution, prepared by dissolving sulphate of copper, the neutral tartrate of potassium and caustic soda, in water, all of them being used in fixed proportions. A little of the clear blue solution thus produced is poured into a test tube and boiled, when no change should take place; now, a few drops of the suspected urine are to be added and heat once more applied, when, if sugar is present, a yellowish-red precipitate will be thrown down. This test acts admirably well, even in unskillful hands, and nothing better could possibly be desired, were it not for certain difficulties, to which we have alluded before, in making and keeping the test solution. Two of the constituents of this test liquid, the neutral tartrate of potassium and the caustic soda, are not found, in a state of purity, outside the large cities, and even in these latter are frequently to be obtained only with difficulty; hence, it becomes impracticable for the great majority of practitioners to use this test at all, from their inability to procure readily the necessary chemicals. But a still greater misfortune in connection with this test, is, that even after the necessary solution has been prepared, it frequently cannot be relied upon, for almost always, in the course of time, it will spoil and become worthless, owing to a reaction that is brought about by the presence of the neutral tartrate of potassium Consequently, this truly beautiful and accurate test becomes almost valueless, except to physicians living in the large cities, where the requisite chemicals can be easily obtained, and the test solution may be as often renewed as necessary.

Some two years since we commenced a series of experiments, with the object of discovering, if possible, chemicals to be substituted for the neutral tartrate of potassium and caustic soda, in Fehling's test solution, which would not decompose upon keeping, and which could be readily obtained whenever needed, and so, while retaining the great simplicity and great delicacy of Fehling's test, at the same time obviate its objectionable features. We soon found that glycerine* and caustic potassa exactly filled these requirements, producing a test liquid, when dissolved in water, along with sulphate of copper, which, even in uneducated hands affords most excellent results, and which two years of experience we think warrants us in saying will not decompose however long it may be kept. Moreover, these substances can be procured in a state of sufficient purity wherever a drug store exists, and, consequently, the test comes within the reach of every practitioner.

---

*We were surprised to find, a short time since, that this use of glycerine has been previously suggested by a foreign chemist, but we were entirely unaware of this fact while making our own investigations. Since, however, the employment of glycerine in testing for sugar is almost unknown in this country, we believe that our suggestions concerning its use will be quite as acceptable as if our proposal for employing it had not been anticipated.

The formula that we ordinarily employ for making up this modified and improved test liquid, is the following:

Take of sulphate of copper, thirty grains; of hydrate of potassium (in sticks), one and a half drachms; of *pure* glycerine, two fluid drachms; and of pure water (distilled is best), six fluid ounces. Divide the water into two nearly equal portions; in one of these parts dissolve the sulphate of copper and the glycerine, and in the other portion the hydrate of potassium. Mix the two solutions and stir until any precipitate which may in the first place be formed is entirely re-dissolved. The test liquid thus prepared may now be poured into a bottle and set aside until needed.

It will be perceived that this solution may be made up with the greatest possible ease, and any physician, in ten minutes of spare time, can procure the necessary chemicals and prepare enough of it to last him a year or two, even if he be frequently called upon to use it in testing for sugar. It will, perhaps, however, be frequently found most advantageous for the physician to send the formula as given above to some druggist and have the test liquid prepared by him, just as if it were a prescription for some medicine. When well made the solution should be a perfectly clear, dark blue liquid, free from sediment, and a little of it boiled in a test tube should undergo no change. During the first week or two after it is prepared a slight reddish precipitate of the sub-oxide of copper (cuprous oxide) will be thrown down, owing to the presence of trifling impurities in the glycerine and caustic potassa employed, but after this no further change will take place in the liquid, however long it may be kept, if the chemicals employed in preparing it were reasonably pure. We have in our possession a quantity of this test solution prepared nearly two years since, and yet it is in as perfect a condition now as the day it was made.

After a quantity of this solution has been prepared, testing for sugar in the urine becomes a matter of the greatest simplicity, and ought not to occasion the physician the slightest trouble. An ordinary sized test tube is to be filled to the depth of half an inch or so with the test liquid, and heat from any convenient source applied until it boils, when no change whatever should ensue. Now, two or three drops of the suspected urine are to be added, the test tube shaken slightly, so as to cause a mixture of the two fluids, and heat once more applied. If sugar is present in any considerable quantity, an abundant precipitate of the yellowish-red sub-oxide of copper (cuprous oxide) will be produced, and this, remaining for a while suspended in the liquid, will make the latter appear almost opaque, by reflected light. If, however, the test solution undergoes no change on thus treating it with two or three drops of the urine under examination, we may be certain that no considerable quantity of sugar is present, but in order to demonstrate its *entire* absence it will be necessary to continue the addition of the urine, until a quantity has been added equal to half the bulk of the test liquid, and then apply heat once more. If any sugar whatever be present it will now show itself by causing a characteristic precipitate of the sub-oxide of copper—it may be, however, only in small quantity. But, if on the contrary, sugar be entirely absent, no change in the test liquid will ensue, except a lightening of the color from the dilution, and the appearance of a white, flocculent deposit of phosphates floating about in the light, greenish-blue liquid. Care should be taken not to mistake this phosphatic deposit for one of the sub-oxide of copper; the former is white and flocculent, the latter yellowish-red and shows no disposition to form flocculi.

Such is the simple method of employing this test liquid, and if in using it an ordinary amount of care be observed, the result may be implicitly relied upon, which certainly can scarcely be said of the other tests for sugar in the urine.

If we wish to determine not merely the presence or absence of sugar, but its quantity also, we may, by the use of the test liquid as prepared above, arrive at very accurate results, employing the volumetric process, such as is used with Fehling's unmodified test solution in making quantitative determinations. But as this method requires considerable apparatus which practitioners seldom possess, and necessitates the expenditure of not a little time and skill, it becomes almost impracticable for ordinary clinical uses. But rough approximate results, which in the majority of cases are all that a physician has practically any need of, can be readily obtained by observing the amount of precipitate that two or three drops of the urine will occasion in a drachm of the test liquid, boiled in a test tube,—an abundant precipitate indicating a large amount of sugar, a slight precipitate a small quantity, and so on. We may by this means compare from day to day, or week to week, the amount of sugar in diabetic urine, and thus judge, somewhat crudely, it is true, of the progress of the disease and of the effects produced by the treatment employed.

We believe that this improved Fehling's test which we have just described somewhat in detail, is possessed of advantageous features found in no other test, and we are confident that almost every physician who will once employ it will be exceedingly loth to return again to any of the previously used methods, none of which can compare with it in simplicity, delicacy, and ease of application.

---

UNIVERSITY OF BERLIN.—According to the *Gazette de Cologne* there was a marked falling-off in the attendance upon the winter course of 1873 and 1874 in the Berlin University, especially noticeable in the medical department. This diminution is attributed to the increased advantages, not only at Leipsig, but also at Halle and many other Prussian universities, and particularly at Gottingen.

DR. GARABETTI (*London Lancet*) has obtained the best results from the administration of enemata of potass. brom., in doses from one-half to two drachms in cases of obstinate vomiting attending the pregnant state; the same drug, also administered in enema, has been very useful in the hands of Dr. Laborde, of Paris, in obstinate vomiting connected with disease of the stomach, liver, and intestines.—*Med. News and Library.*

